# An Ultrasound Classification of Anterior Talofibular Ligament (ATFL) Injury

**DOI:** 10.2174/1874325001711010610

**Published:** 2017-07-31

**Authors:** Yehua Cai, Shengkun Li, Shiyi Chen, Yinghui Hua, Jielin Shan

**Affiliations:** 1 Department of sonography, Huashan Hospital, Fudan University, Shanghai, China; 2 Department of Sports Medicine and Arthroscopy Surgery, Huashan Hospital, Fudan University, Shanghai, China

**Keywords:** Ankle, Anterior talofibular ligament, Injury, Ultrasound, Classification

## Abstract

**Background:**

To develop a classification of ATFL injury based on the ultrasonography.

**Methods:**

The ultrasound images of 560 cases that had chronic ankle instability were studied from May 2012 to May 2015. All the patients accepted ultrasonography to type the ATFL injury.

**Results:**

The ATFL injuries could be divided into six subtypes based on ultrasound imaging: I: Intact ligament; II: Injury at the fibular side; III:Injury at the talar side; IV: Tear at the midsubstance; V:Ligament absorbed; VI:Combined injury.

**Conclusion:**

Ultrasound could be used to evaluate and type the injury of ATFL. This kind of classification could be helpful in the preoperative decision of ATFL procedure.

## 1 INTRODUCTION

Anterior talofibular ligament (ATFL) injury is generally accepted as one of the most important reasons for chronic ankle instability. For the patients who do not respond to conservative treatment, operation aimed at ATFL injury is recommended. There are several kinds of procedures to deal with ATFL injury: 1) Broström procedure, which imbricates the ligament and makes a midsubstance repair [1] ; 2) An open or arthroscopic procedure to repair the ligament at fibula side [1-3] ; 3) A procedure to repair the ligament at talus side [4]; 4) A procedure with arthroscopic ATFL shrinkage [2]; 5) An open or arthroscopic procedure with ATFL reconstruction (non-anatomic or anatomic) [1, 2, 5]. But until now there are no exact criteria for a surgeon to decide which procedure should be used preoperatively. Most surgeons still decide the operative strategy based on clinical diagnosis pre-operatively or the findings during the operation, and the result of operation might be affected.

Ultrasound is one of the most sensitive tools to diagnose the injury of ligament and other soft tissue and quantify the ligament integrity [6]. In this study, we developed a new classification of ATFL injury based on ultrasound imaging to help surgeons select the procedure when dealing with the ATFL injury.

## 2 MATERIALS AND METHODS

Total 560 patients who had unilateral chronic symptomatic ankle instability accepted ultrasound examination from May 2012 to May 2015, including 394 male patients and 166 female patients. The median age of the patients was 25 years (range 13– 56 years). The criteria to select patients were as follows: 1) The patients had a history of chronic ankle twist injury for more than 3 months; 2) The patients had symptoms of instability such as giving away or twist injury at least two times; 3) The patient did not accept operation at the same limb before.

All the sonographic procedures were performed by the same radiologist with use of an ALT HDI 5000 US unit (Philips Medical Systems, Bothell, WA, USA), and a wide frequency linear array transducer with center frequencies from 5 to 17 MHz was used.

All the ultrasound examinations were performed by the same radiologists (the authors in this article) with the same technique described in our previous article [7]. Patients were in supine position with the ankle in a maximal inversion and flexion position. The transducer was placed along the long axis of ATFL to show the whole ligament. After that the transducer was rotated 90° to show the short axis of the ATFL. Then, the ATFL of contralateral side was also examined.

The ligament was defined as a thin, anisotropic, hyperechoic, rectilinear band at the long axis, while in transverse scans it was defined as a small ovoid structure filled with uniform hyperechoic dots. The scar was defined as uniform hyperechoic area, while the capsular was defined as an anisotropic, cross-arrangement band with a weaker signal comparing to ligaments [7].

**Fig. (1):**
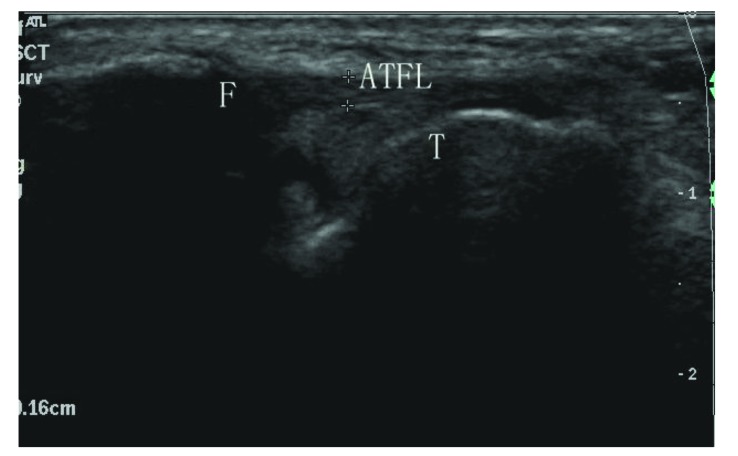
Sonogram shows a normal anterior talofibular ligament (type I). ATFL: anterior talofibular ligament; F: fibula; T: talus.

**Fig. (2):**
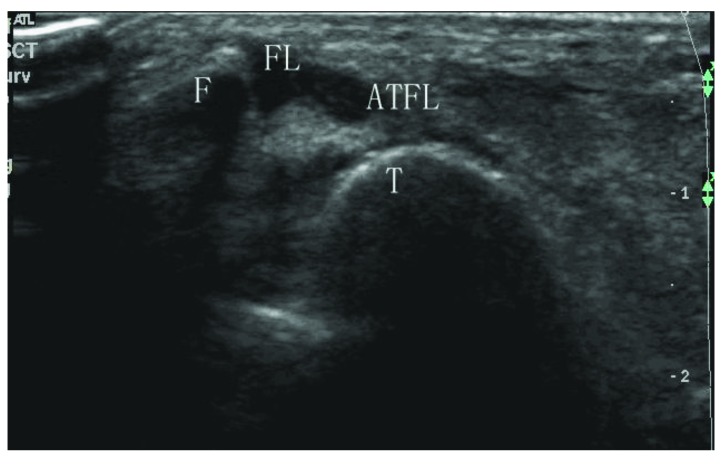
Sonogram shows a total tear at the fibular side (type IIB). ATFL: anterior talofibular ligament; F: fibula; T: talus; FL: fluid.

**Fig. (3):**
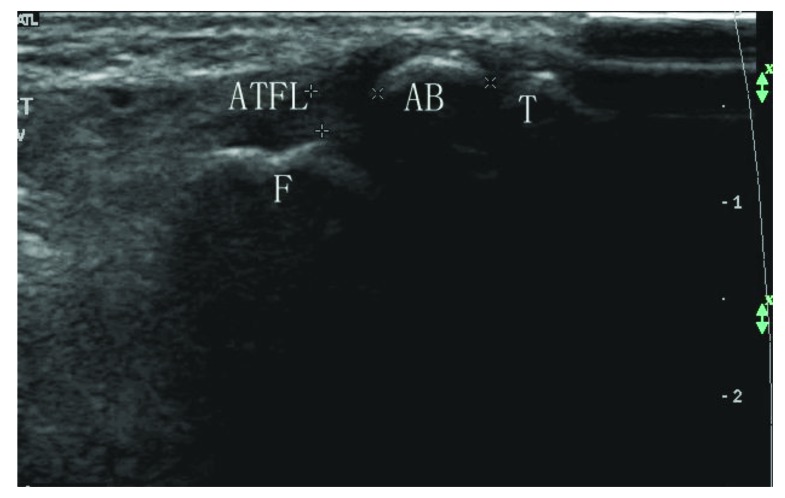
Sonogram shows a hyperechoic shadow (diameter > 0.5cm) in the remanent ligament at the fibular side (type IIC). ATFL: anterior talofibular ligament; F: fibula; T: talus; AB: avulsion bone

**Fig. (4):**
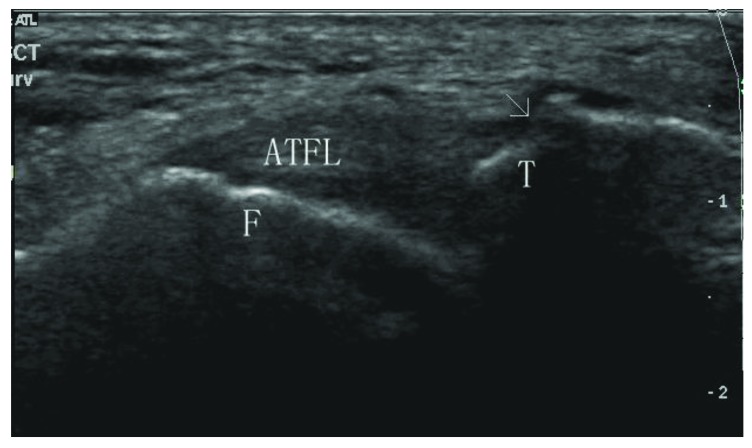
Sonogram shows a total tear at the talar side (type IIIB). ATFL: anterior talofibular ligament; F: fibula; T: talus; Arrow: ligament tear at the talar side.

**Fig. (5):**
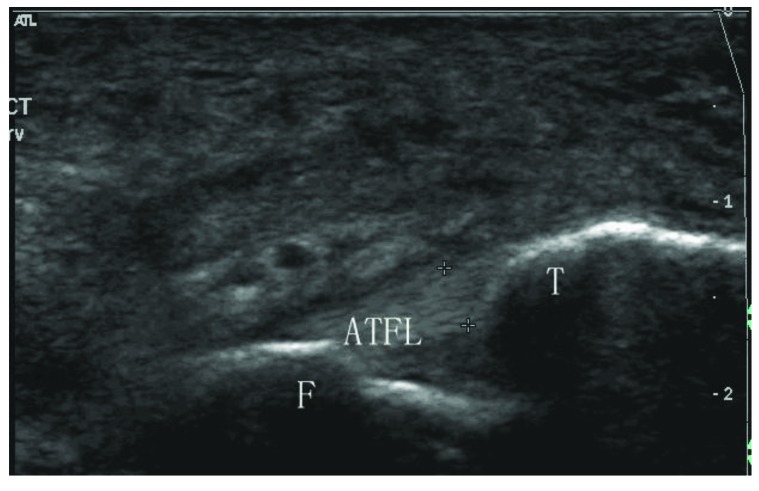
Sonogram shows the ligament thicker than 2.4 mm wide (type IV). ATFL: anterior talofibular ligament; F: fibula; T: talus.

**Fig. (6):**
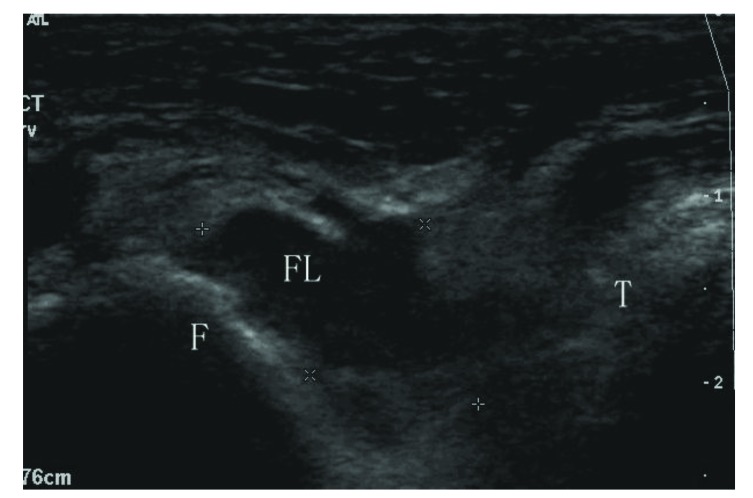
Sonogram shows the ligament fiber has been absorbed and could not be seen. (type V). F: fibula; FL: fluid; T: talus.

**Fig. (7):**
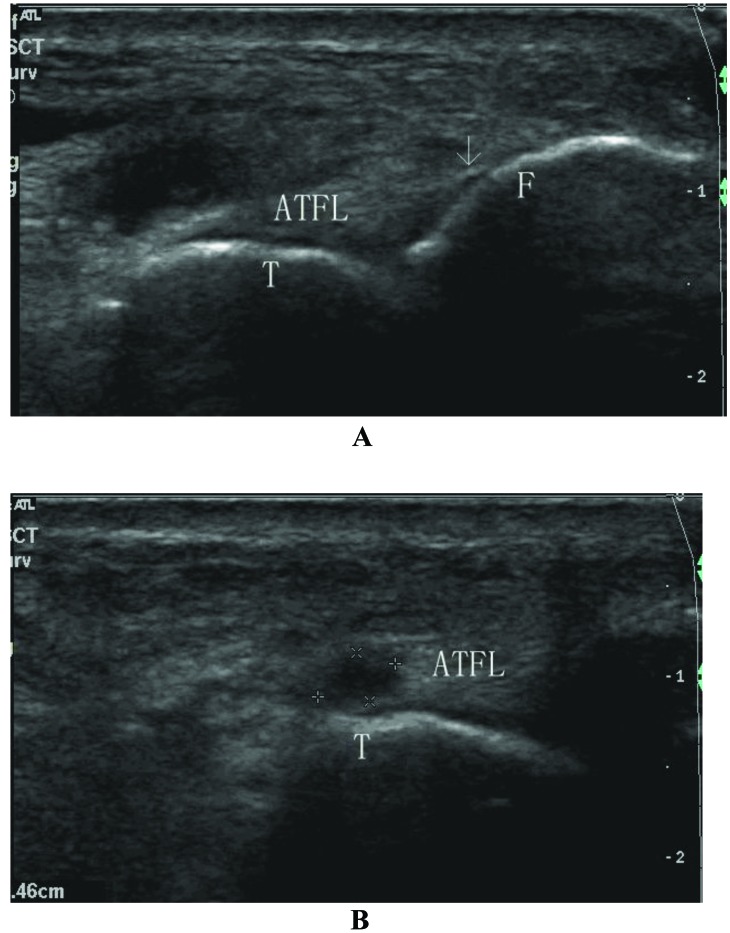
Sonogram shows the ligament injury at **A**) the fibular side (calcification) and **B**) the talar side (tear) (type VI). ATFL: anterior talofibular ligament; F: fibula; T: talus; Arrow: calcification at the fibular side; Star: ligament tear at the talar side.

According to the following criteria, a classification of ATFL injury was suggested (Figs. **1**-**7**):

Intact ligament: Continuous hyperechoic fibers are found from the anterior part of the lateral malleolus (fibular side) to the anterior part of the talus (talar side). The texture of the ligament is clear. The bone surface where the ligament inserts is smooth.

Injury at the fibular side:

A: Partial ligament tear at the fibular side;

IIB: Total ligament tear at the fibular side: there is a total interruption of the ligament fibers at the fibular side, but ligament fibers could be found at the talar side. More than 50% of the ligament remains.

C: Fibular avulsion fracture: 1) Hyperechoic shadow (diameter > 0.5cm) could be seen in the remanent ligament at the fibular side; or 2) irregularities of the fibular surface and X-ray show the avulsion fracture (diameter > 0.5 cm).

Injury at the talar side:

IIIA: Partial ligament tear at the talar side;

IIIB: Total ligament tear at the talar side: there is a total interruption of the ligament fibers at the talar side, but ligament fibers could be found at the fibular side. More than 50% of the ligament remains.

Tear at the midsubstance: The ligament fibers could be found at both sides, but 1) the ligament is still relaxed when the foot is in plantar flexion and the forefoot is oriented slightly medially; or 2)It is thicker than 2.4 mm wide or exceeds 20% than the width of the opposite ligament. The ligament texture is fuzzy and hypoechoic although continuous hyperechoic fibers are found from the fibular side to the talar side; or 3)some small hypoechoic regions could be found in the ligament.

V: Ligament absorbed: The residual ligament is less than 50% of the whole ligament.

Combined injury: More than one interruption (partial or total) of the ligament fibers could be found at either side or midsubstance region, but more than 50% of the ligament remains.

## 3 RESULTS

All the patients were divided into six types. The percentage of each type was calculated as (Table **1**). The type IIinjury accounted for the largest part in all the parts, which was 29.64%, followed by type IV(24.64%),type I(20.54%), type V(11.61%), type III(9.29%), and type VI(4.29%).

## 4 DISCUSSION

Until now, it is still puzzling for a surgeon to diagnose and evaluate the injury of ATFL. It has been stated that the routine methods, including physical examination, stress X-ray, arthrography or MRI, are not reliable to evaluate the chronic ATFL injury [1, 8-10]. While the ultrasound has been confirmed to be a reliable and accurate method to evaluate chronic ATFL injury [7, 11, 12].

For a torn ligament, the ideal treatment is to repair the ligament in situ where the ligament was torn. We design the classification on account of the following assumption: The result of US could be an evidence for surgeon to select the operative procedure for ATFL injury. In this classification, we include ligament texture, ligament continuity, location where ligament torn, degree of ligament thickening, degree of residual ligament, large strong echo zone and cortical continuity as the criterion of the classification. Other abnormalities such as irregularities of the talar bone, small high level echo zone in the ligament, small level echo zone beside the ligament were not included into the classification because they would not affect the selection of the operation procedure.

Another consideration of the design is that the type III injury doesn’t have a subtype of IIIC. The reason is that the avulsion fracture of talus is seldom seen in the clinical situation [13]. More important, it would not affect the selection of operative procedure.

For the type injury, one of the criteria is the thickness of the ligament. The width of the ATFL is thought to be about 2 mm, so, a ligament thicker more than 20% of this standard is thought to be a thick ligament [14].

The thick ligament might be attributed to the scar because of the healing of the ligament. But, the ligament might become thick because of the retraction when the ligament tears at the either side. So, if there is injury at the either side, a type II or III injury but not type IV injury is considered.

A ligament is often injured at one site, but recurrent twists could induce the injury at multiple sites. In our research, the type VI injury is seldom but still could be seen. The characteristic is that there are always 2 partial tears at this type.

Until now, lots of surgeons would like to use the same procedure to deal with all the patients. Our research shows that in the patients with ATFL injury, three types of injuries (I, II and IV) compose majority of the injuries (more than 70%). But no type could be than 50%. This result indicates that there is no a single procedure could deal with all the patients.

Based on the results of the evaluation of ultrasound, we recommend the following operation strategy: 1) Injury of type I, typeIIA, Type IIIA: Conservative treatment is recommended. 2) Type IIB injury: We suggest to employ an open or arthroscopic ligament repair at fibula side; 3) Type IIC injury, we advise the fixation of the bone fragment, or the procedure to repair the ligament to a roughened fibula after removal of the bone fragment; 4) Type IIIB injury, we suggest to employ an open or arthroscopic ligament repair at talar side; 5) Type IV injury, we suggest the Broström procedure, arthroscopic ATFL shrinkage or ligament repair with augmentation at fibular side;6) Type V injury, we suggest ATFL reconstruction; 7) Type VI injury, depending on the degree of the injury, a repair procedure at the site where the injury is most severe, ATFL reconstruction or conservative treatment can also be selected depending on the degree of the injuries.

There are also some shortcomings in this study. At first, the sonographic evaluation for chronic ATFL injury is not a golden standard, which would make the proportion of the sub-types ligament injury different from the real condition. While the previous studies have showed it had high accuracy and reliability even comparing to the operative detection [7, 12], we believe the classification based on the sonographic evaluation would help the clinician to make the strategy of the treatment for chronic ankle instability. Secondly, there is no clinical result to support whether the new classification guided treatment is better than the present operation strategy. A further prospective study is needed to confirm the clinical value of this classification. Thirdly, we did not employ the dynamic ultrasound to evaluate the ATFL injury in this study because it had not been routinely used in our clinic. We believe it might improve the accuracy of the examination and we would make it as a routinely method in the future [15]. Furthermore, the type of calcaneofibular ligament injury is not included in this study because a classification including two ligament injuries would be too complex. In addition, some studies have questioned the necessity of repairing CFL when it was injured [16, 17]. So we prefer to type the CF injury in the other classification system.

## CONCLUSION

In this study, we classified the injury of ATFL into six types based on the results of the ultrasound. This classification might be helpful in the decision of the operation strategy for chronic ankle instability.

## Figures and Tables

**Table 1:** Data of the Patients who accepted US examination.

Type	I	II			III	IV	V	VI	Total
		A	B	C					
Cases	115(20.54%)	48(8.57%)	98(17.50%)	20(3.57%)	52(9.29%)	138(24.64%)	65(11.61%)	24(4.29%)	560(100%)
L/R	47/68	28/20	57/41	6/14	20/32	76/62	42/23	11/13	287/273
M/F	87/28	35/13	76/22	16/4	41/11	81/57	40/25	18/6	394/166

L/R: Left side /Right side. M/F: Male/Female.
